# Duration of infectiousness and correlation with RT-PCR cycle threshold values in cases of COVID-19, England, January to May 2020

**DOI:** 10.2807/1560-7917.ES.2020.25.32.2001483

**Published:** 2020-08-13

**Authors:** Anika Singanayagam, Monika Patel, Andre Charlett, Jamie Lopez Bernal, Vanessa Saliba, Joanna Ellis, Shamez Ladhani, Maria Zambon, Robin Gopal

**Affiliations:** 1 Virus Reference Department, Public Health England, Colindale, United Kingdom; 2 These authors contributed equally; 3 Data and Analytical Services, Public Health England, Colindale, United Kingdom; 4 Immunisation and Countermeasures, Public Health England, Colindale, United Kingdom

**Keywords:** SARS-CoV-2, COVID-19, coronavirus, infectiousness, respiratory virus, public health policy

## Abstract

Severe acute respiratory syndrome coronavirus 2 viral load in the upper respiratory tract peaks around symptom onset and infectious virus persists for 10 days in mild-to-moderate coronavirus disease (n = 324 samples analysed). RT-PCR cycle threshold (Ct) values correlate strongly with cultivable virus. Probability of culturing virus declines to 8% in samples with Ct > 35 and to 6% 10 days after onset; it is similar in asymptomatic and symptomatic persons. Asymptomatic persons represent a source of transmissible virus.

Since the emergence of coronavirus disease (COVID-19) at the end of 2019, rapid tracing and isolation of confirmed cases and close contacts with restrictions on social movement have played an important role in controlling onward spread of the virus. Understanding the duration of infectiousness in persons who test positive for severe acute respiratory syndrome coronavirus 2 (SARS-CoV-2) is critical to developing evidence-based public health policies on isolation, contact tracing and return to work. Virus detection by reverse transcription-PCR (RT-PCR) from respiratory samples is widely used to diagnose and monitor SARS-CoV-2 infection and, increasingly, to infer infectivity of an individual. However, RT-PCR does not distinguish between infectious and non-infectious virus. Propagating virus from clinical samples confirms the presence of infectious virus but is not widely available, requires biosafety level 3 facilities, and the results are not timely to inform public health actions. The aim of this work was to understand how RT-PCR detection relates to cultivable virus, which can be used as a proxy for infectiousness and can inform and support decisions on infection control.

## Kinetics of viral RNA detection from the respiratory tract

Upper respiratory tract (URT) samples from persons with suspected COVID-19 were tested at the national respiratory virus reference laboratory at Public Health England to support routine clinical care and surveillance activities during the COVID-19 pandemic. Samples included nose, throat, combined nose-and-throat and nasopharyngeal swabs, or nasopharyngeal aspirates; the majority were taken by clinical staff but some were self-sampled nose swabs.

**Figure 1:**
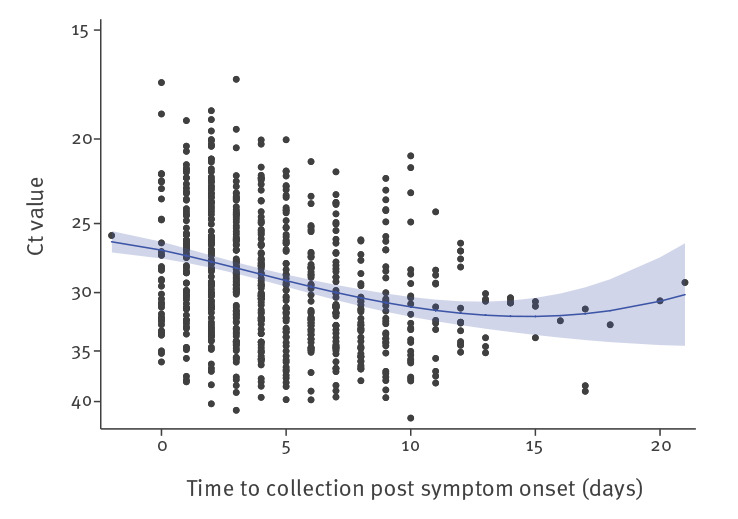
Detection of SARS-CoV-2 by RT-PCR targeting the RdRp gene, England, January–April 2020 (n = 754)

In the first 3 months of the COVID-19 pandemic in the United Kingdom (UK) (late January to early April 2020), we received 754 URT samples from 425 symptomatic cases that tested positive for SARS-CoV-2 by RT-PCR targeting the RNA-dependent RNA polymerase (RdRp) gene [1] and that had a clear record of the dates of symptom onset and sample collection. These samples were collected as part of the First Few 100 surveillance study described in Boddington et al. [2]. Using RT-PCR cycle threshold (Ct) values as a semiquantitative measure of SARS-CoV-2 viral load identified that the level of SARS-CoV-2 RNA in the URT was greatest around symptom onset, steadily decreased during the first 10 days after illness onset and then plateaued (Figure 1). In the first week after symptom onset (days −2 to 7), geometric mean (GM) Ct was 28.18 (95% confidence interval (CI): 27.76–28.61). In the second week (days 8 to 14), GM Ct was 30.65 (95% CI: 29.82–31.52; p < 0.001 compared with week 1) and after 14 days, GM Ct was 31.60 (95% CI: 31.60–34.49; p = 0.01 compared with week 1). There was no significant difference in Ct values between days 8–14 and after 14 days (p = 0.49). 

Ct: cycle threshold.

To capture the nonlinear relationship between days post symptom onset (days) and Ct value, a fractional polynomial model was used, indicating predictors days^2^ and days^2^ln(days). These were fitted in a random intercept regression model with ln(Ct value) as the outcome variable. Analysis accounted for multiple samples from the same individuals. The random intercept for individuals was not statistically significant, providing no evidence for dependencies within person, thus each individual sample was treated as being independent.

## Isolation of infectious virus from respiratory samples

Virus culture was attempted from 324 URT samples (from 253 cases) that tested positive for SARS-CoV-2 by RT-PCR. Samples were obtained from a range of clinical scenarios including community and healthcare worker surveillance, symptomatic persons tested as part of the early epidemic response and samples acquired in outbreak investigations. Selection of asymptomatic cases was through swabbing of contacts or facility/family/household testing in the context of outbreak investigations; we cannot be certain of their date of exposure or start of infection. Vero E6 cells were inoculated with clinical specimens and incubated at 37 °C, 5% CO_2_. Cells were inspected for cytopathic effect daily up to 14 days. Presence of SARS-CoV-2 was confirmed by SARS-CoV-2 nucleoprotein staining by enzyme immunoassay on infected cells. Cultivable virus was isolated from 133 (41%) samples (from 111 cases).

Median Ct of all 324 samples was 31.15 (interquartile range (IQR): 27.50–33.86; range: 17.47–41.78). Some 233 cases (92%) were classified as non-severe (asymptomatic or mild-to-moderate) and 20 (8%) had severe illness (requiring intensive care admission and/or fatal). There was no difference in Ct values between those with asymptomatic (median Ct = 31.23; IQR: 28.21–32.97), mild-to-moderate (median Ct = 30.94; IQR: 27.08–34.57) or severe (median Ct = 32.55; IQR 28.39–33.66) illness (p = 0.79). A stratified comparison of the severe cases over time showed a similar result as described above: Ct values were lower (higher viral load) in week 1 than week 2. There were 62 samples from 61 asymptomatic cases and no difference in culture positivity rate was observed: 21 of 62 samples from asymptomatic individuals vs 112 of 262 samples from symptomatic individuals (estimated odds ratio (OR) = 0.66; 95% CI: 0.34–1.31; mixed effects logistic regression model, p = 0.23).

## Relationship between Ct value and virus isolation

**Figure 2:**
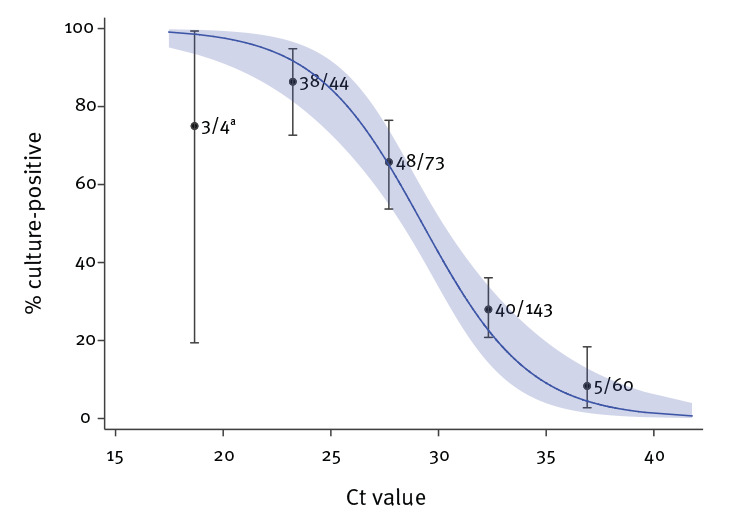
Relationship between RT-PCR Ct value and culture positivity in mixed effects logistic regression analysis, SARS-CoV-2, England, January–May 2020 (n = 324)

We observed a strong relationship between Ct value and ability to recover infectious virus. The estimated OR of recovering infectious virus decreased by 0.67 for each unit increase in Ct value (95% CI: 0.58–0.77) (Figure 2). Virus propagation was successful from five of 60 samples with Ct > 35; all five were from symptomatic cases and none had severe illness. The estimated probability of recovery of virus from samples with Ct > 35 was 8.3% (95% CI: 2.8%–18.4%).

Ct: cycle threshold.

^a^ Wide confidence intervals due to small sample size.

## Relationship between ‘symptom to test’ interval and virus isolation

**Figure 3:**
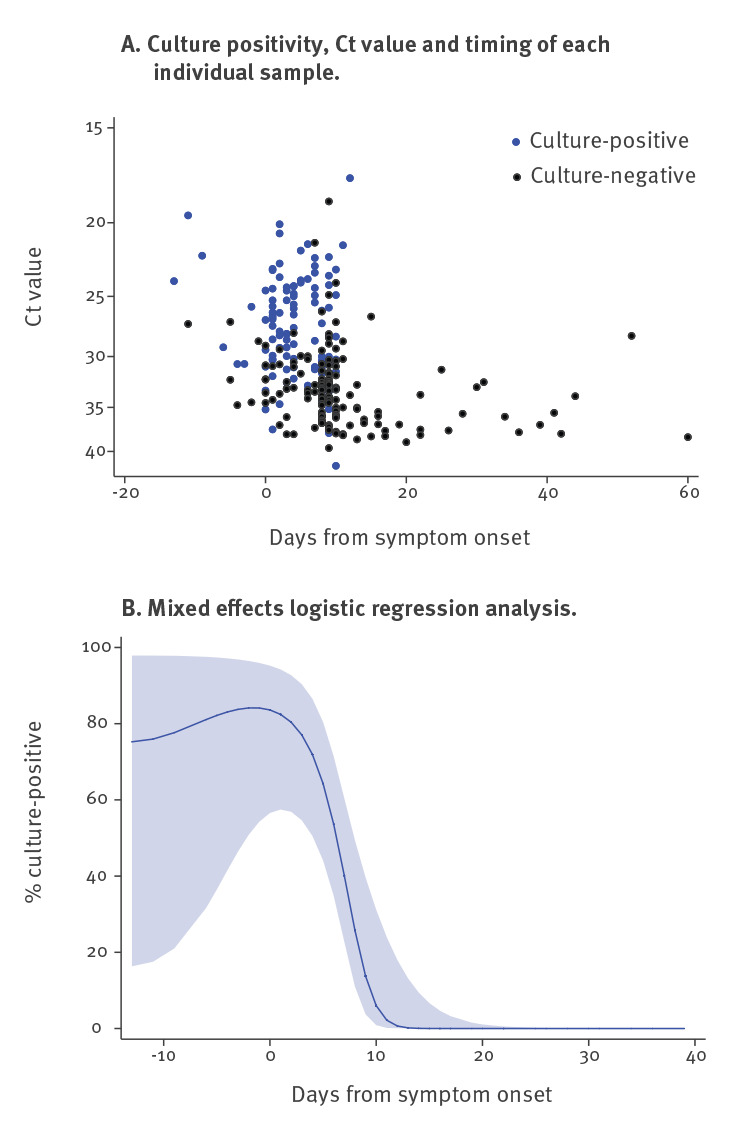
Relationship between culture positivity and time between symptom onset and sample collection, SARS-CoV-2, England, January–May 2020 (n = 246)

There were 246 samples from 176 symptomatic cases where the date of symptom onset was known, of which 103 (42%) samples from 81 cases were culture-positive. Detection of cultivable virus peaked around the time of symptom onset (Figure 3). Median duration of virus shedding as measured by culture was 4 days (IQR: 1–8; range: −13 to 12, with symptom onset dates based on symptom recall). The culture positivity rate was significantly higher during week 1 than week 2 (74% vs 20%; p = 0.002). Ten days after symptom onset, the probability of culturing virus declined to 6.0% (95% CI: 0.9–31.2%) (Table 1). Where cases were followed up within outbreak investigations, 13 individuals who were asymptomatic at the time of sampling developed symptoms within 14 days of sampling and were classified as presymptomatic, of whom seven were culture-positive. Regression analysis indicates that presymptomatic samples were at least as likely to be culture-positive as samples taken during symptomatic phases.

Ct = cycle threshold.

**Table 1:** Estimated percentage of SARS-CoV-2 samples culture-positive 7–15 days after symptom onset, England, January–May 2020 (n = 121)

Day post symptom onset	Estimated^a^ percentage culture-positive (95% CI)	N (observed number tested)	R (observed number culture-positive
7	40.1 (22.8–60.4)	14	10
8	25.8 (11.0–49.4)	33	9
9	13.7 (3.7–39.6)	34	10
10	6.0 (0.9–31.2)	23	6
11	2.2 (0.2–23.9)	6	1
12	0.7 (0.0–17.9)	3	1
13	0.2 (0.0–13.1)	4	0
14	0.03 (0.0–9.4)	2	0
15	0.006 (0.0–6.7)	2	0

CI: confidence interval.

^a^ From mixed effects logistic regression model.

More than half the samples (n = 130, 53%) tested (from 91 cases) were received more than 7 days after symptom onset and 21% of those (27 samples from 18 cases) were culture-positive; none of the 91 patients had severe illness or were immunosuppressed. Most of these late culture-positive samples (25/27) were taken between 8 and 10 days after symptom onset.

## Comparison of virus detection by age group

There was no significant difference in Ct values (p = 0.12) or culture positivity (p = 0.63) from URT samples received across the different age groups, although this dataset included few children younger than 16 years. The proportion of asymptomatic cases was similar across age groups, except for 81–100 year-olds who were more likely to be asymptomatic than the other age groups (p = 0.006, cluster-adjusted logistic regression) (Table 2). There was no difference in the proportion of asymptomatic cases between males and females, with an estimated OR of 0.86 (95% CI: 0.46–1.90; p = 0.63).

**Table 2:** Comparison of virus detection and presence of symptoms, by age group, England, January–May 2020 (n = 324)

Age group in years	Number of cases	Ct value		Virus isolation		Asymptomatic cases	
		Geometric mean	95% CI	Estimated % culture-positive	95% CI	%	95% CI
0–20	14	28.81	26.50–31.33	57.8	26.7–83.8	14.3	3.0–47.3
21–40	81	30.81	29.77–31.90	43.2	30.7–56.5	17.5	10.0–28.9
41–60	140	30.83	30.03–31.65	37.7^a^	27.8–48.7	13.6	8.6–20.8
61–80	40	29.87	28.42–31.38	41.3	24.4–60.5	17.5	7.8–34.6
81–100	49	29.09	27.84–30.41	32.1	18.8–49.2	40.8	27.4–55.7

CI: confidence interval; Ct: cycle threshold.

Estimated geometric mean Ct value, proportion culture-positive and proportion asymptomatic from random intercept linear, random intercept logistic regression and cluster adjusted logistic regression models.

^a^ Total excluding one case because one sample was cytotoxic on cell culture.

## Discussion

Readouts from semiquantitative RT-PCR using Ct values provide a valuable proxy for infectious virus detection and may help to inform decision-making on infection control. This study adds to the evidence base on duration of infectiousness following mild-to-moderate COVID-19, demonstrating that infectious virus can persist for a week or more after symptom onset, declining over time. At 10 days after symptom onset, in line with current guidance from the World Health Organization [3] and the UK [4] on release from isolation, probability of culturing virus declines to 6%. The findings concur with smaller studies that identified infectious virus shed for 8 or 9 days [5-9] and others demonstrating correlation between Ct value/viral load and cultivable virus [5,9-11]. Strengths include the comparatively large size of this dataset, inclusion of a large proportion (> 50%) of samples taken more than 7 days after symptom onset and that all analysis was performed in a single laboratory. Van Kampen et al. reported more prolonged detection of cultivable virus from 23 hospitalised cases, for up to 20 days after symptom onset [10]. However, their cohort included mostly lower respiratory tract samples from patients with more severe disease including nearly one in five who were immunocompromised, which is unlikely to be representative of the general population. Taken together with data presented here, the results of Van Kampen et al. indicate that more prolonged excretion of infectious virus could be associated with severe disease or an immunocompromised state.

This study identified that Ct values and the presence of infectious virus were similar in samples from asymptomatic and presymptomatic persons, compared with those who were symptomatic, and is one of the first reports of virus isolation from cases who remain completely asymptomatic. The findings suggest that asymptomatic and presymptomatic persons do represent a source of potentially transmissible virus. Extensive data on cultivable virus from asymptomatic or presymptomatic individuals are lacking, with one outbreak investigation in a care home reporting detection of cultivable virus in one asymptomatic and 17 presymptomatic cases [8]. Although we saw a higher proportion of asymptomatic cases in the age group 81–100 years, the reasons and significance of this are unclear. It may reflect sampling bias from care home outbreaks. However, it could also reflect real differences in response to infection in this age group (e.g. lower response to fever, lower reporting of subjective symptoms in this age group).

Of note, recall bias may affect the interpretation of timing of virus detection in relation to symptom onset, particularly in elderly patients and those presenting with atypical symptoms. Duration and cessation of symptoms is also not well recorded. For asymptomatic cases, the time when infection was acquired is not known. A further limitation is that this dataset comprises real-world data and subjects were not sampled systematically; therefore, there may be bias in the timing of sampling related to the clinical scenario. Finally, the sensitivity of virus propagation from clinical samples is dependent on laboratory expertise, cell lines and protocols used, and may be affected by sample quality, storage and transport conditions, meaning it is difficult to directly compare data between laboratories described in other literature.

## Conclusion

Based on the real-world data described here, we recommend that infection control measures for persons with mild-to-moderate COVID-19 be particularly focussed immediately after onset of symptoms and retained for 10 days. Asymptomatic and presymptomatic persons are likely to be a source of infectious virus. Detection of cultivable SARS-CoV-2 from URT samples is valuable as a proxy for infectiousness; however, as the human infectious dose remains unknown, the significance of low titres of infectious virus for human-to-human transmission remains uncertain. Correlation with observational epidemiological data analysing known infector–infectee pairs is required to fully understand the dynamics of infectiousness and viral transmissibility.
