# Correction for Euro Surveill. 2020;25(32)

**DOI:** 10.2807/1560-7917.ES.2021.26.7.210218c

**Published:** 2021-02-18

**Authors:** Eurosurveillance editorial team

**Affiliations:** 1 European Centre for Disease Prevention and Control (ECDC), Stockholm, Sweden

In the article ‘Duration of infectiousness and correlation with RT-PCR cycle threshold values in cases of COVID-19, England, January to May 2020’ by Singanayagam et al., published on 13 August 2020, an explanatory comment was added on 18 February 2021 to the footnotes of Figures 1 and 3.

